# Coproducing data-driven organizational safety with patients: development and cognitive testing of a multisetting patient-reported safety concern tool

**DOI:** 10.1093/intqhc/mzaf056

**Published:** 2025-06-24

**Authors:** Abubakar Sha’aban, Anna Torrens-Burton, Denitza Williams, Andrew Carson-Stevens, Adrian Edwards, Lee Joseph, Natalie Joseph-Williams

**Affiliations:** Health and Care Research Wales Evidence Centre, Division of Population Medicine, Cardiff University, Heath Park, Cardiff CF14 4YS, United Kingdom; PRIME Centre Wales, Division of Population Medicine, Cardiff University, Heath Park, Cardiff CF14 4YS, United Kingdom; Health and Care Research Wales Evidence Centre, Division of Population Medicine, Cardiff University, Heath Park, Cardiff CF14 4YS, United Kingdom; PRIME Centre Wales, Division of Population Medicine, Cardiff University, Heath Park, Cardiff CF14 4YS, United Kingdom; Health and Care Research Wales Evidence Centre, Division of Population Medicine, Cardiff University, Heath Park, Cardiff CF14 4YS, United Kingdom; PRIME Centre Wales, Division of Population Medicine, Cardiff University, Heath Park, Cardiff CF14 4YS, United Kingdom; NHS Wales Performance & Improvement, Oldfield Road, Bocam Park, Pencoed, Bridgend, CF35 5LJ, United Kingdom; Health and Care Research Wales Evidence Centre, Division of Population Medicine, Cardiff University, Heath Park, Cardiff CF14 4YS, United Kingdom

**Keywords:** patient safety, patient-reported concerns, tool development, focus groups, cognitive interviews, healthcare improvement

## Abstract

**Background:**

Patient safety is a critical aspect of healthcare, with patients often being the first to notice safety concerns. However, traditional reporting mechanisms have limitations, and many patients may not report safety issues due to fear of repercussions or lack of clarity in existing systems. There is a growing need for tools that enable patients to report safety concerns easily and effectively. This study aimed to undertake preliminary development and cognitive testing of a Patient Reported Safety Concern Tool, designed to capture a broad range of patient safety issues across various healthcare settings that could enhance quality of care and foster continuous safety improvement.

**Methods:**

A two-phase, qualitative study was conducted in Wales, virtually through online platforms (Zoom or MS Teams) between January and September 2023. In Phase 1, 26 adults (aged 25–54, 23.1% female) participated in three online focus groups recruited through purposive sampling. In Phase 2, 10 additional participants (aged 25–84, 70% female) were purposively sampled for online cognitive interviews. Participants were eligible if they were 18+ and had accessed healthcare within the past 6 months. Individuals with professional expertise in patient safety were excluded. Data were analysed using qualitative content analysis in NVivo 12. A coding framework was developed inductively and iteratively refined.

**Results:**

Focus group participants preferred the term ‘safety concern’ over terms like ‘incident’ or ‘event,’ as it was more relatable and inclusive of both physical and emotional harm. Feedback led to refinements in item clarity, such as extending the recall period to 6 months and rewording prompts for detail. Cognitive interviews confirmed that version 2.0 was easy to understand and relevant. Minor adjustments were made, including extending the recall period to 12 months and adding ‘ambulance services’ as a setting. The final version, 3.0, demonstrated high content and face validity, with participants expressing a strong willingness to complete the tool if distributed routinely.

**Conclusion:**

The Patient Reported Safety Concern Tool was co-developed with public participants and refined through cognitive testing, demonstrating strong content and face validity. Participants felt confident the tool would help identify safety concerns not captured through conventional systems. Future work will focus on validating the tool in wider populations, understanding barriers to completion, and integrating it into existing patient safety learning systems to inform actionable safety improvements.

## Introduction

Identifying and analysing patient safety incidents is critical for continuous learning and improvement in healthcare systems, as emphasized by the World Health Organization’s (WHO) Global Patient Safety Action Plan 2021–30 [1]. Many countries have mandated the routine capture of such incidents through national quality and safety delivery plans, ensuring data analysis drives meaningful organizational learning [2]. While patients’ roles in improving safety are increasingly recognized [3, 4], their insights are often not integrated into healthcare systems as effectively as organizationally reported data. To address this gap, it is essential to develop mechanisms that make patient-reported data both accessible for patients and actionable for organizations, moving beyond mere incident metrics to meaningful safety insights.

Patients provide unique perspectives on safety incidents, including near-misses or concerns not captured in organizational reporting systems or medical records [5–8]. This complementary role is vital for a holistic understanding of harm in healthcare, as neither organizational reports nor patient accounts alone represent a ‘gold standard’ [8]. Patient-reported data can serve multiple purposes, including enhancing accountability, highlighting systemic risks, and fostering bilateral learning between patients and healthcare organizations [3]. However, achieving these goals requires tools that effectively capture and translate patient-­reported safety concerns into actionable insights.

The COVID-19 pandemic provided an unprecedented opportunity to explore patient-reported safety concerns across diverse healthcare settings. During the pandemic, a seven-item Patient Reported Safety Concern Tool (version 1.0) was developed by a working group comprising patients, health services researchers, and clinicians. This tool was designed to assess the occurrence, nature, seriousness, avoidability, and impact of safety concerns. It was incorporated into the UK COVID-19 Public Experiences (COPE) study [9], a longitudinal mixed-methods study conducted in a UK community setting. The COPE study aimed to identify determinants of health behaviour over the course of the pandemic, guided by the Capability, Opportunity, Motivation—Behaviour model. A total of 13 604 participants completed the COPE survey across three time points, with 1363 reporting patient safety concerns [10]. This demonstrates the tool’s potential to capture real-time safety issues during a rapidly evolving public health crisis.

The tool was subsequently used in Evaluation of the Shielding Initiative in Wales (EVITE Immunity) study [11], which examined the impact of a UK government policy introduced in 2020 advising clinically extremely vulnerable individuals to self-isolate for 12–16 weeks. As the Shielding Initiative was implemented without prior evidence of its effects on health or behaviour, the tool was employed to capture patient-reported safety concerns arising from this intervention—such as unintended consequences related to disrupted access to healthcare and essential services, as well as emotional and psychological distress. While these applications demonstrated the tool’s utility, its expedited development process necessitated further testing and refinement to enhance its comprehensiveness, clarity, and feasibility. These insights informed the current study’s iterative development process.

Patient-reported outcome and experience measures (PROMs and PREMs) offer valuable frameworks for capturing safety concerns, but their integration into learning healthcare systems remains limited [12]. Despite promising feasibility results for the validated primary care patient measure of safety (PC PMOS) [13], we were not aware at the time of any validated multisetting patient-reported safety measures, and none that captured free-text qualitative data. Addressing this gap, the current study aims to undertake preliminary development and validation of the Patient Reported Safety Concern Tool (version 1.0), ensuring its content and face validity and its potential for embedding within organizational safety learning systems. This tool (version 1.0) was rapidly adapted from the Patient Incident Reporting Tool, originally developed for research into patient involvement in safety within the hospital setting [7, 14]. Following initial scoping review of literature of existing patient-reported safety tools, our objectives were to (i) describe what ‘patient safety’ means to the public, (ii) explore content validity of the preliminary tool, (iii) determine face validity with the target population, and (iv) produce a revised version of the Patient Safety Concern Tool (version 3.0) for further testing, development, and validation in diverse patient populations and healthcare settings, and consultation with key stakeholders in learning organizations to explore how it can be embedded into the learning systems.

## Methods

The methods were informed by COSMIN guidelines for developing patient-reported measures [15] and conducted in two phases. This qualitative study took place virtually in Wales between January and September 2023, using online platforms (Zoom or MS Teams). Phase 1 focused on content validation and refinement to create version 2.0, while Phase 2 involved cognitive testing to assess face validity and refine the tool further, resulting in version 3.0.

### Participants

Eligible participants were adults (18+) residing in Wales who had accessed healthcare services within 6 months. Exclusion criteria included professional expertise in patient safety or communication impairments precluding participation. Recruitment targeted diverse demographics through social media, charities (e.g. The Patients Association), and a study webpage, which provided detailed information and a screening questionnaire. In Phase 1, 47 individuals responded to the screening questionnaire, and 26 were purposively selected to ensure diversity in age, gender, ethnicity, and educational attainment. In Phase 2, 28 responded, and 10 were purposively sampled using the same criteria. The screening questionnaire confirmed eligibility, captured demographic information, and supported purposive sampling. Participants in Phase 1 (focus groups) and Phase 2 (cognitive interviews) were from separate cohorts. Focus groups lasted ∼90 min, while cognitive interviews ranged from 45 to 60 min. Eligible participants were contacted for consent and scheduling of focus groups or cognitive interviews.

### Data collection

#### Phase 1: Focus groups—item refinement and development

Conducted online via Microsoft Teams between January and March 2023, focus groups were video recorded and facilitated by researchers (A.S./N.J.W.) using a semi-structured topic guide. The topic guide was co-developed with a public partner, informed by prior literature on co-production [16], patient engagement and safety reporting [17, 18], and pilot tested with two public contributors unaffiliated with the study to refine question clarity, structure, and flow. Discussions explored participants’ understanding of patient safety and their feedback on version 1.0 of the tool, previously used in the COPE [9] and EVITE Immunity [11] studies for comprehension, completeness, usefulness, and feasibility. Participants also assessed alternative items from other published tools [7, 19–21] to identify gaps and evaluate relevance. Researchers practiced reflexivity by maintaining reflective notes and meeting regularly to consider how their perspectives might shape data collection and interpretation. Feedback informed revisions, resulting in version 2.0 of the tool (Table 3).

**Table 3.** Changes to the patient safety concern tool following Phase 1 focus groups and Phase 2 cognitive interviews

Item No.	Version 1.0 Item and response options	Phase 1 focus group suggested changes	Version 2.0 Item and response options	Phase 2 cognitive interview suggested changes	Version 3.0 Item and response options
1	While trying to access or receive NHS or private healthcare during the [coronavirus pandemic]^a^, have you, or someone you care for, experienced something that you thought was a ‘safety concern’? *Safety concerns can be any event or situation where a patient or other people (e.g. relatives, visitors, NHS staff) might have been harmed while accessing NHS care. This includes events or situations where nobody was not actually harmed but they could have been, or where someone could be harmed in the future if the concern is not addressed.* Yes—when using NHS services Yes—when using private healthcare services^a^ No/Not applicable—I have not used NHS services since April 2021 Don’t know Rather not say	This item is modified to reflect 6 months Definition of safety concern should appear before question 1 to aide understanding of the question. Enhance definition by highlighting that harm could include emotional and physical harm.	*Safety concerns can be any event or situation where a patient or other people (e.g. relatives, visitors, NHS staff) might have been harmed while accessing NHS care. This includes events or situations where nobody was not actually harmed but they could have been, or where someone could be harmed in the future if the concern is not addressed.* While trying to access or receive NHS healthcare during the last 6 months have you, or someone you care for, experienced something that you thought was a ‘safety concern’? Yes—when using NHS services No/Not applicable—I have not used NHS services in the last 6 months Don’t know Rather not say	Recall period changed from 6 to 12 months to align with typical routine NHS follow-up intervals. Range of harm added to definition.	*Safety concerns can be any event or situation where a patient or other people (e.g. relatives, visitors, NHS staff) might have been harmed while accessing NHS care. Harm could be physical, psychological, social, or financial.* *It can also include events or situations where nobody was harmed but they could have been, or where someone could be harmed in the future if the concern is not addressed.* While trying to access or receive NHS healthcare during the last 12 months, have you, or someone you care for, experienced something that you thought was a ‘safety concern’? Yes—when using NHS services No/Not applicable—I have not used healthcare services in the last 12 months Don’t know Rather not say
2	In which month (or months) did the safety concern(s) happen? *Please tick all that apply* November 2021 December 2021 January 2022 February 2022 March 2022 April 2022	The original response options were selected as the survey was specific to the pandemic period. Discussions focused on preferred maximum timeframe for recalling incidents. Participants recommended 6 months as the maximum reference period, due to recall bias.	Question: When did the safety concern happen? *Please tick all that apply* 6 months ago 3–5 months ago 1–2 months ago less than a month ago	In line with changes to Item 1, recall period changed to 12 months.	Question: When did the safety concern happen? *Please tick all that apply* less than a month ago between 1 and 3 months ago between 3 and 6 months ago between 6 and 12 months ago other (please state) unsure
3	On a scale from 1 (not serious at all) to 10 (extremely serious), how serious do you think your safety concern was? *How serious was the event?* *1 2 3 4 5 6 7 8 9 10*	No changes required.	On a scale from 1 (not serious at all) to 10 (extremely serious), how serious do you think your safety concern was? *How serious was the event?* *1 2 3 4 5 6 7 8 9 10*	No changes required.	On a scale from 1 (not serious at all) to 10 (extremely serious), how serious do you think your safety concern was? *How serious was the event?* *1 2 3 4 5 6 7 8 9 10*
4	In which healthcare setting(s) did the safety concern(s) take place? *Please tick all that apply.* COVID-19 testing services COVID-19 vaccination services GP services (e.g. GP, nurse appointment, health visitor) A&E Routine outpatient services Inpatient services Midwifery and maternity District nurse Optician Pharmacist Dentist NHS 111 service Other please specify	Response option updated to reflect the postpandemic period e.g. ‘COVID-19 vaccination services’ was modified to ‘vaccination services’ and ‘COVID-19 testing services’ removed.	In which healthcare setting(s) did the safety concern(s) take place? *Please tick all that apply.* Vaccination services GP services (e.g. GP, nurse appointment, health visitor) A&E Routine outpatient services Inpatient services Midwifery and maternity District nurse Optician Pharmacist Dentist NHS 111 service Other (please specify)	‘Ambulance services’ added as a response option A&E acronym expanded to full word (‘Accident & Emergency) and acronym Suggested to focus the item to one ‘event’ or safety concern. Changed from ‘please tick all that apply’ to ‘choose the most appropriate option from the following list’.	Where did the safety concern happen? *Choose the most appropriate option from the following list.* Ambulance services Accident and Emergency (A&E) Routine outpatient services Inpatient services Vaccination services Midwifery and maternity District nurse Optician Pharmacy Dentist NHS 111 service GP services (e.g. GP, nurse appointment) Other (please specify)
5	What did the safety concern(s) relate to? *Please tick all that apply.* Vaccination Diagnosis of your problem Access to the NHS service you needed Tests or procedures that were performed (e.g. blood tests, scans Medication or treatment Delay or cancellation of treatment for pre-existing condition Communication between you and the healthcare professional(s) Communication and co-ordination between different healthcare professionals Concerns specific to the coronavirus outbreak (e.g. personal protective equipment) Information that was provided to you Other	Choices are updated to match the postpandemic period (response option ‘concerns specific to the coronavirus outbreak…’ was removed).	What did the safety concern(s) relate to? *Please tick all that apply.* Vaccination Diagnosis of your problem Access to the NHS service you needed Tests or procedures that were performed (e.g. blood tests, scans Medication or treatment Delay or cancellation of treatment for pre-existing condition Communication between you and the healthcare professional(s) Communication and co-ordination between different healthcare professionals Information that was provided to you Other	No changes required.	What did the safety concern(s) relate to? *Please tick all that apply.* Vaccination Diagnosis of your problem Access to the NHS service you needed Tests or procedures that were performed (e.g. blood tests, scans Medication or treatment Delay or cancellation of treatment for pre-existing condition Communication between you and the healthcare professional(s) Communication and co-ordination between different healthcare professionals Information that was provided to you Other
6	In a few sentences, please tell us a bit more about what happened when you experienced the safety concern and the impact it has had on you or the person you care for. *Free text response—no character limit*	A few sentences indicated that people could not provide detail if they wanted to. Recommended to be rephrased to ‘please tell us a bit more about what happened….’	Please tell us a bit more about what happened when you experienced the safety concern and the impact it has had on you or the person you care for *Free text response—no character limit*	Changes suggested to question wording to improve clarity and readability. Changed to ‘please tell us a bit more about what happened and what impact it has had on you or the person you care for’.	Please tell us a bit more about what happened and what impact it has had on you or the person you care for. *Free text response—no character limit*
7	Question: Do you think it would have been possible to have stopped this safety concern from happening? Definitely yes Probably yes Probably not Definitely not Don’t know	No changes required.	Question: Do you think it would have been possible to have stopped this safety concern from happening? Definitely yes Probably yes Probably not Definitely not Don’t know	Changes suggested to question wording to improve clarity and readability. Changed to ‘Do you think anything could have been done to stop it from happening?’ (response options did not change).	Do you think anything could have been done to stop it from happening? Definitely yes Probably yes Probably not Definitely not Don’t know
8	N/A	This item on patients’ experience of various forms of harm was adapted from a study by Ricci-Cabello, Avery [25], as acknowledged by certain participants who believed it would effectively encompass their first-hand experiences related to safety concerns.	Do you think you have experienced any of the following types of harm as a result of the healthcare provided in an NHS healthcare setting you visited in the last 6 months? *5-point Likert scale (not at all; hardly any; yes, somewhat; yes, a lot; yes, extreme)* Harm to your physical health Harm to your mental health Increased limitations in doing your usual social activities Increased healthcare needs Increased personal needs Increased financial needs	In line with changes to Item 1, recall period changed to 12 months.	Do you think you have experienced any of the following types of harm as a result of the healthcare provided in an NHS healthcare setting you visited in the last 12 months? *5-point Likert scale (not at all; hardly any; yes, somewhat; yes, a lot; yes, extreme)* Harm to your physical health Harm to your mental health Reduced abilities to do your social activities Increased healthcare needs Increased personal needs Increased financial needs

a Private healthcare services were included in version 1.0 as this was within remit for the COPE study. Subsequent versions were amended to only include NHS settings; the target setting for the tool.

#### Phase 2: Cognitive interviews

From July to September 2023, individual cognitive interviews using a ‘think aloud’ method were conducted online using Microsoft Teams or Zoom. Researchers (A.B./A.T.B.), both trained in qualitative methods, employed a concurrent ‘think aloud’ approach [22], in which participants verbalized their thought processes as they read and responded to each item in version 2.0. A semi-structured interview script (co-developed [16] with a public partner, informed by best practices in cognitive interviewing [23]) was used to guide the interviews and pilot tested with two public contributors not involved in the main study. The script ensured consistency across interviews while allowing flexibility to probe emerging insights. Interviews focused on assessing face validity, including item clarity, comprehensibility, and overall tool format. Researchers maintained detailed field notes and practiced reflexivity throughout, discussing their own interpretations and assumptions during analysis. Participant feedback guided refinements incorporated into version 3.0 of the tool.

### Data analysis

#### Focus groups

Focus group transcripts were transcribed verbatim and analysed using qualitative content analysis [24] in NVivo 12. An inductive approach was taken, with initial open coding applied to capture participants’ own language and interpretations. The coding framework was developed by A.S. through repeated readings of one transcript and expanded to include emergent codes based on recurring patterns across groups. Codes were then grouped into categories related to patient safety definitions, item comprehension, and feasibility. This framework was then reviewed and refined through discussion with N.J.W. to ensure alignment with the study’s aims. Transcripts were reviewed iteratively, with the refined coding framework applied across all transcripts to explore how themes developed and diverged between focus groups. Regular team meetings supported ongoing interpretation, reflexivity, and rigour in analysis.

#### Cognitive interviews

Interview data underwent similar content analysis in NVivo 12 to assess the face validity of version 2.0. Researchers (A.S./N.J.W.) identified themes related to item clarity and usability. Emerging themes were validated through discussion and incorporated into version 3.0.

## Results

**Figure 1:**
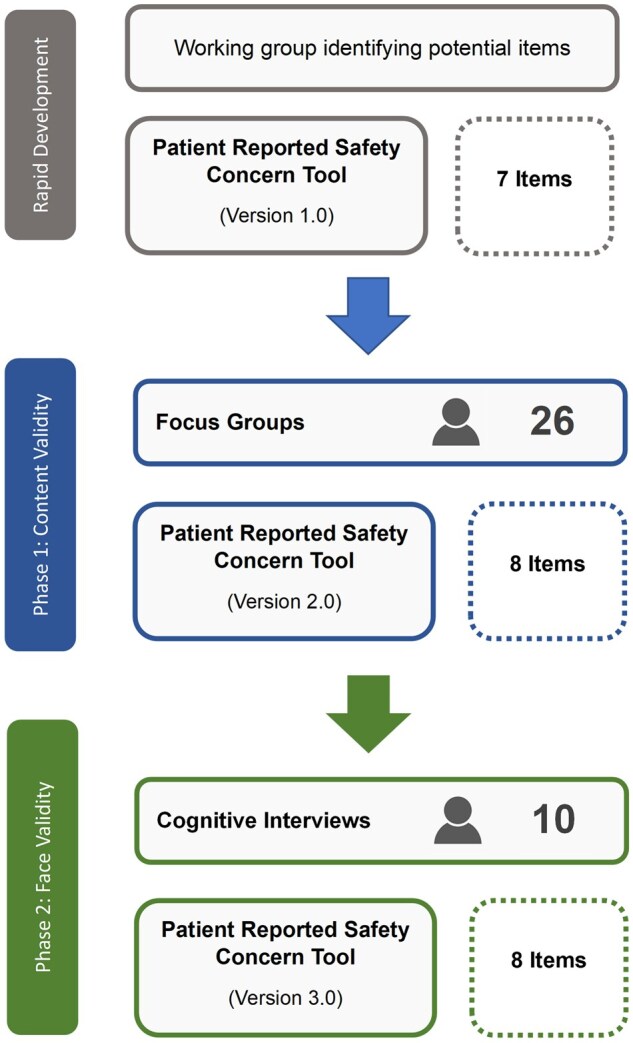
Summary of content validity (Phase 1: focus groups) and face validity (Phase 2: cognitive interviews) stages of the Patient Reported Safety Concern Tool

A summary of participants, tool refinements, and versions are available in Fig. 1.

### Participants

Demographic characteristics of focus group and cognitive interview participants are presented in Table 1.

**Table 1.** Demographic characteristics of focus group (*n* = 26) and cognitive testing (*n* = 10) participants

	Focus groups	Cognitive testing
	Number (%)	Number (%)
Ethnicity		
Welsh, English, Scottish, Northern Irish, or British	7 (26.9)	4 (40)
Black, African, Caribbean, or Black British	6 (23.1)	1 (10)
White and Black African/Caribbean	8 (30.8)	1 (10)
African/Caribbean	4 (15.4)	1 (10)
Roma	1 (3.8)	
Arab		1 (10)
Indian		1 (10)
Bangladeshi		1 (10)
Age (years)		
18–24	4 (15.4)	
25–34	16 (61.5)	2 (20)
35–44	5 (19.2)	5 (50)
45–54	1 (3.8)	
55–64		1 (10)
65–74		1 (10)
75–84		1 (10)
Gender		
Male	17 (65.4)	2 (20)
Female	6 (23.1)	7 (70)
Prefer not to say	3 (11.5)	1 (10)
Highest educational level		
University level qualification	16 (61.5)	7 (70)
School or college leaver qualification	9 (34.6)	3 (30)
No formal qualifications	1 (3.8)	

### Phase 1: focus groups—item refinement and development

There were 26 participants across three focus groups (*n* = 9, *n* = 9, *n* = 8). Ages ranged from 25–54 years (mean = 32) and 23.1% were female; three participants chose not to disclose their gender. Focus groups were 60–90 min in duration.

### Phase 2: Cognitive interviews

There were 10 cognitive interview participants. Ages ranged from 25–84 years (mean = 46.5) and 70% were female; one person preferred not to disclose their gender. Interview length ranged between 30 and 60 min.

### Key findings

#### Phase 1: Focus groups

Participants shared their views on what ‘safety concerns’ meant to them (Table 2) across the three focus groups (‘F’ indicates focus group number and ‘P’ indicates participant identifier). There was a range of responses from ‘how well patients are taken care of’ [F1P3, male] to ‘avoidance of unintended or unexpected harm to people’ [F1P8, male]. Key themes emerging across the responses included being *‘*cared’ for, ‘protecting’ patients, patient ‘wellbeing’, preventing ‘harm’, ‘fairness’, and ‘equity’. All participants felt it was an essential part of healthcare, e.g. ‘patient safety is kind of very important and shouldn’t be neglected in any way, because it ensures the general care and wellbeing of a patient’ [F1P2, male].

**Table 2.** What ‘patient safety’ means to the focus group participants

What ‘patient safety’ means (direct quotes from participants)	Focus group/participant/gender
We’re looking at the general overview of what it means to care for someone who is sick	F1P2, male
Patient safety just means a way of preventing harm to the patient	F1P4, male
Avoidance of unintended or unexpected harm to people during the provision of their healthcare	F1P8, male
Operation safety is an aspect where [the] operation experiences a very good outcome, there is equity in our system, and it's being treated fairly. The patient has self-confidence on every strategy he has confided in the facility in which he found himself…this makes him or her feel safe…	F2P19, male
Patient safety is the prevention of healthcare errors and the elimination of the admission and mitigation of patients’ injury caused by health error…so when a patient is given proper treatment, and the treatment also goes along with the patient’s illnesses	F2P10, male
Patient safety means the system used to help the patients…keeping them safe from harm, other possible complexities that come up within the healthcare	F2P11, male
It can be emotional safety and also physical safety	F2P13, female
Keeping patients safe from health problems	F2P14, female
Patient safety means like the general wellbeing of patients and the communication between patient and healthcare workers	F2P16, male
How we can make sure our patients have a good living and are able to really communicate very well with our healthcare workers to be able to get efficient and sustainable healthcare services	F2P17, male
For me, as a person, and as for my family, I feel safe in any medical facility, when I realize that I’ve been treated fairly, and everybody there makes me feel okay, and everything is accurate on time, there is real reliability	F2P19, male
I think safety concern means the care and the well-being of patients	F3P23, male
To me safety concern means how the patient has been cared for, their well-being… it really has to do with your safety and them being in a safe space in a healthy, safe, safe space	F3P20, male

In version 1.0, the term ‘safety concern’ was chosen over the term ‘incident’, commonly used in clinical safety processes, to describe patient safety events. Participants reviewed alternatives (e.g. incident, event, harm, episode) and agreed that ‘safety concern’ was the most suitable. They found it understandable, easy to relate to, and all-encompassing. Some participants noted that they liked the term ‘concern’ because it had a bidimensional meaning, indicating that the healthcare system cared and was also concerned about patients and their safety.

Patient safety concern means a lot to me, because it shows that there are some concerns that the NHS is having for the patient [F3P20, male]

Other participants shared COVID-19 pandemic-specific unsafe experiences, including staff shortages, poor hygiene, inadequate infection control, bias, and racism, noting that the term ‘concern’ accurately captured such experiences. Further discussions emphasized the clarity and comprehensiveness of tool items. Participants felt that the items used lay terminology that were easy to understand and complete, with refinements detailed in Table 3.

For Item 2 (when the safety concerned happened), Phase 1 participants highlighted that an ideal recall interval depends on individual circumstances, concern severity, and personal significance. A 6-month timeframe was deemed optimal for most, minimizing recall bias. Item 3 (severity) was universally regarded as clear and effective, with participants noting its utility in helping organizations prioritize actionable concerns.

Response options in Item 4 (setting) and Item 5 (what the concern related to) were seen as comprehensive, encompassing most healthcare settings and issues. However, participants recommended removing pandemic-specific options to ensure long-term relevance. The multiresponse format of Item 4 raised concerns about linking issues to specific settings. To streamline analysis and enhance organizational utility, participants suggested revising this to a single-response format, focusing on the most relevant setting.

All participants valued the additional information that would be captured in free text in Item 6 (asking them to describe the concern), e.g.:

This question will help expressing more information in the way it’s supposed to, it’s more like a space for vital information [F2P13, female]

However, the phrasing ‘in a few sentences’ might indicate that a thorough and detailed description of the concern was unnecessary or not desired. As a result, patients might only provide a superficial overview, which could lack the depth needed to adequately understand the concern and be useful for organizational learning. Therefore, Item 6 was rephrased to ‘please tell us a bit more about what happened’.

During the focus groups, we presented alternative versions of items that were included in version 1.0 and additional items that were not included (drawn from the literature review). Participants noted that the current survey does not make it clear that patient safety concerns could be physical and emotional in nature:

But it didn't really specify the kind of harm because, when you talk about harm, it could be physical harm. It could be emotional harm. So, I believe on something should be added to it [F1P1, male]

To address this, an additional item identified in our literature review [25] was included in version 2.0 (Item 8, Table 3).

Overall, participants reported that they would be comfortable completing this survey routinely, and the majority preference for distribution was an online survey sent via email; although some participants recommended raising awareness of the survey in local community settings (e.g. supermarkets, barber shops, religious venues).

#### Phase 2: Interviews—cognitive testing

All participants found the majority of version 2.0 items easy to understand with only minimal changes suggested. Results from the ‘Think Aloud’ cognitive interviews are presented in Supplementary Appendix S1, which shows the number of participants who understood the main item statements and response options. The suggested changes were minor and included sentence shortening, enhancing clarity, and extending the recall period from 6 to 12 months to align with routine National Health Service (NHS) or General Practice (GP) follow-up intervals (Table 3). Amendments were made to Items 1, 2, and 8 to reflect the extended 12-month recall period. ‘Ambulance services’ was added as an additional response option for Item 4. The wording of Items 6 and 7 and one response option from Item 8 were identified as lengthy and overly complex; these were changed to enhance clarity. Items 3 and 5 remained unaltered. The refined tool (version 3.0) integrating these recommendations is presented in Table 3.

## Discussion

### Statement of principal findings

This study presents the refinement and preliminary testing of a Patient Reported Safety Concern Tool (version 3.0), with a focus on its content and face validity. Through insights gained from focus groups and cognitive interviews, we enhanced the tool’s clarity, relevance, and comprehensibility. Participants affirmed the tool’s usability, demonstrating a willingness to complete it to contribute to organizational learning. They viewed patient safety as an integral part of healthcare delivery rather than a distinct activity, emphasizing the interplay between safety processes and care provision. Key findings from Phase 1 (focus groups) included broad consensus on the relevance and need for the tool, but there were disagreements regarding the clarity and wording of specific items. Some participants emphasized the need for more emotional or relational aspects of safety to be captured, while others focused on procedural and clinical safety concerns. Differences sometimes emerged within focus groups, reflecting variation in individual healthcare experiences, literacy levels, and expectations [26]. These inconsistencies were addressed by refining item wording for clarity and adding an item for generalizability. In Phase 2 (cognitive interviews), participants generally found version 2.0 clear and usable but flagged areas where phrasing could still be improved.

Participants expressed motivation to use the tool beyond merely reporting concerns, recognizing its potential to inform safety improvements. However, this motivation was contextualized by the study’s research setting, necessitating further investigation into real-world implementation across diverse populations.

Our findings also highlighted the concept of ‘voiceable concerns’, with participants expressing uncertainty about when and how to report certain experiences. This subjective process (determining whether an event was significant enough to warrant reporting) suggests that supportive resources and clear definitions will be critical for successful implementation.

### Interpretation within the context of the wider literature

This study also offers important insights into how the public conceptualizes ‘patient safety’. Participants in our study described safety as a multidimensional construct that encompassed physical, emotional, interpersonal, and systemic elements. Safety was not seen merely as the absence of harm, but as the presence of care, respect, clear communication, fairness, and confidence in health systems. This broad conceptualization aligns with earlier findings that patients frequently embed safety within their wider experience of care [17, 27–29]. They perceive safety as relational and embedded in trust, timeliness, and reliability, rather than only defined by technical outcomes.

This contrasts with traditional clinical definitions of patient safety [30], which tend to focus on preventable adverse events and system failures. Our findings add nuance to the literature by reaffirming that public understandings are shaped by lived experience and a holistic view of care, including emotional safety and equity. Some participants also showed awareness of systemic aspects of safety, suggesting that the public can engage with both micro- (individual care) and macrolevel (organizational and structural) dimensions of safety when given the appropriate tools and language. These perspectives support the design of safety concern tools that reflect the full breadth of what patients experience and value.

This study supports existing evidence that patient-reported data can provide unique insights into safety concerns not captured by organizational systems [6, 8]. The findings align with research highlighting patient-reported barriers to engagement, such as fear of repercussions and power imbalances, which can discourage reporting even in urgent safety scenarios [31–33]. In this context, the anonymous nature of the Patient-Reported Safety Concern Tool may help to mitigate such barriers, supporting existing evidence that anonymity can increase willingness to report safety concerns [34, 35].

Addressing these barriers requires an understanding of the broader factors influencing patient engagement, including task complexity, healthcare professional attitudes, and contextual factors such as busy work environments or perceived importance of the concern [32]. In addition to enhancing the tool itself, leveraging organizational processes known to support engagement with patient feedback—such as accreditation requirements, quality improvement cycles, and existing safety monitoring systems—could maximize the tool’s impact. Prior research has shown that integrating patient feedback into structured safety systems, like the PRASE intervention, can improve staff responsiveness and organizational learning [36–38]. Embedding the Patient-Reported Safety Concern Tool within these established processes may therefore offer a practical and sustainable route for implementation.

The challenge of distinguishing between ‘negative experiences’ and ‘voiceable concerns’ is also well-documented [39]. Healthcare workers themselves often struggle with subjective interpretations of safety concerns, suggesting the need for clear guidance and support for patients navigating similar decisions [40]. Integrating frameworks such as Systems Engineering Initiative for Patient Safety extended model (SEIPS 2.0) and the Healthcare Complaints Analysis Tool can help systematize the analysis of patient-reported data, providing actionable insights for organizational learning.

### Strengths and limitations

A key strength of this study is the iterative development process, which ensured that the tool aligns with patient perspectives and enhances its relevance and accessibility. The inclusion of focus groups and cognitive interviews with a diverse sample further strengthened the tool’s applicability across different healthcare settings. Additionally, this work contributes to the growing body of research [41–43] that underscores the importance of patient-reported data in identifying safety concerns that may otherwise go unnoticed by organizational systems. The anonymous nature of the Patient-Reported Safety Concern Tool may also help mitigate commonly reported barriers to patient engagement in safety reporting, such as fear of blame, repercussions, or power imbalances [31–33]. This aligns with previous findings suggesting that anonymity increases patient or healthcare professionals’ willingness to raise safety concerns [34, 35].

The exploration of ‘voiceable concerns’ during tool refinement is an additional strength, as it provides insight into how subjective decision-making processes can influence patient reporting behaviour and may inform targeted support strategies to facilitate reporting. However, some limitations must be acknowledged. The study was conducted with highly motivated participants in the recovery phase of the disruptive pandemic, who were engaged in the testing and refining the tool. This could have inflated willingness and perceived ease of tool completion. Further work is required to explore the reality of patients completing the tool outside of the research context. Furthermore, broader testing with ‘less heard’ patients, such as individuals with low literacy or limited digital access, is necessary to ensure that the tool is inclusive and equitable. Finally, the long-term utility and integration of the tool into routine safety learning systems remain to be demonstrated.

### Implications for policy, practice, and research

Embedding patient-reported tools into routine care aligns with ‘policy’ trends towards person-centred healthcare systems [44] and quality frameworks emphasizing timely, safe, and effective care [2]. Policymakers should prioritize the integration of such tools into existing safety strategies to complement organizational reporting systems and ensure alignment with overarching healthcare goals. Embedding patient-reported tools into routine care aligns with current policy trends towards person-centred healthcare systems and quality frameworks that emphasize timely, safe, and effective care.

From a ‘practice perspective’, successful implementation of the tool requires careful consideration of several factors. First, patient burden must be minimized by addressing common barriers, such as time constraints, complexity, and potentially unclear utility of the data collected. It is essential to identify preferred modes of delivery, timing, and the level of support required for effective tool use. Second, inclusive design must accommodate diverse populations by ensuring the tool is accessible to individuals with varying literacy levels, digital access, and cultural perspectives. Tailored approaches for underrepresented groups will be critical in achieving equitable data collection. Third, the tool’s data must integrate seamlessly into existing safety reporting mechanisms, aligning with established terminology and workflows [45]. Collaboration with learning organizations and safety teams will be essential to achieve this goal.

‘Future research’ should focus on further validation, including construct validity, relationship to incident reporting (distinguished from ‘concerns’), reliability, and validation with underrepresented groups to ensure it is generalizable and inclusive. Additional studies are needed to investigate how patients distinguish between negative experiences and safety concerns, which will inform guidance and support for their reporting decisions. Drawing on findings from studies of PROMs and PREMs [46], future development should also prioritize reducing response burden while ensuring meaningful data collection. Leveraging artificial intelligence and deep learning methods could further enhance the scalability and efficiency of analysing patient-reported data, enabling the identification of actionable insights on a larger scale.

### Implications for routine safety learning systems

Developing a valid and feasible tool is only the first step; its successful implementation requires that the data collected move beyond basic metrics, such as incident frequency and severity, to provide actionable insights into the causes and prevention of safety concerns. Tools like the PMOS [47] and PC PMOS [7] have demonstrated how patient-reported data can help identify latent conditions and error-producing factors that contribute to safety incidents in both secondary and primary care settings. These approaches may offer valuable lessons for enhancing the depth and utility of the Patient-Reported Safety Concern Tool. Collaborations with learning teams in NHS Wales and other organizations across the UK will be instrumental in exploring how the tool could be used to sit alongside and complement current organizational patient safety learning and integrate into existing strategies and processes [(e.g. routine patient experience PROMs/PREMS, ongoing work with Patient Advice and Liaison Service, Safety and Learning Network, digital systems (e.g. Civica Experience Platform), and patient portals]. Understanding the needs of organizational stakeholders will guide refinements to data collection strategies, ensuring that the tool aligns with safety goals and is both usable and informative.

Consistency in terminology, such as the use of terms like ‘concerns’, ‘incidents’, and ‘complaints’, is also crucial to enhance data interpretability and application. Ongoing work analysing free-text data from earlier tool iterations in the COPE [9] and EVITE studies [11] has demonstrated the richness of patient-reported insights [10]. Applying frameworks like SEIPS 2.0 [48] and patient safety incidents (PISA) [45] has shown potential for identifying system-level improvements. Future analyses with larger samples and advanced methodologies, including automated coding, will further refine these approaches and potentially enhance the tool’s utility.

## Conclusions

We have refined the Patient Reported Safety Concern Tool (version 3.0), demonstrating content and face validity and identifying opportunities for further development and validation. Participants expressed motivation to contribute to safety learning, emphasizing the tool’s potential to provide actionable insights for healthcare organizations. Future work will address barriers to completion, explore patient decision-making processes around ‘voiceable’ safety concerns, and optimize the tool’s integration into routine systems. These efforts will advance co-produced, data-informed safety systems that prioritize patient voices, ultimately enhancing healthcare quality and safety.

## Supplementary Material

mzaf056_Supplementary_Data

## Data Availability

The datasets generated and analysed during the current study are not publicly available due to the sensitive nature of the data and the confidentiality agreements with participants. However, de-identified data may be made available upon reasonable request to the corresponding author, subject to approval by the relevant ethics committee and in compliance with institutional and data protection regulations.
