# Supplementary material for: Coproducing data-driven organizational safety with patients: development and cognitive testing of a multisetting patient-reported safety concern tool
Source: Int J Qual Health Care. 2025 Jun 24;37(3):mzaf056. doi: 10.1093/intqhc/mzaf056 (PMC12246782; doi:10.1093/intqhc/mzaf056)
Supplement: mzaf056_Supplementary_Data [file mzaf056_supplementary_data.zip › Appendix 1_Cognitive interview responses.docx]

**Appendix 1-Responses to Safety Concern Tool (version 2.0) items during the “Think Aloud”**

| **Item Number** | **Questions Easy to Understand** | | **Suggested Changes to main item question*** | | **Suggested Changes to Response Options*** | |
| --- | --- | --- | --- | --- | --- | --- |
|  | Yes | No | Yes | No | Yes | No |
| Item 1 | 7 | 3 | 3 | 7 | 3 | 7 |
| Item 2 | 10 | 0 | 0 | 10 | 3 | 7 |
| Item 3 | 10 | 0 | 0 | 10 | 0 | 10 |
| Item 4 | 8 | 2 | 2 | 8 | 3 | 7 |
| Item 5 | 10 | 0 | 0 | 10 | 0 | 10 |
| Item 6 | 8 | 2 | 2 | 8 | NA | NA |
| Item 7 | 9 | 1 | 1 | 9 | 0 | 10 |
| Item 8 | 10 | 0 | 0 | 10 | 4 | 6 |

*****The suggested changes are presented in Table 3 and explained in the Key Findings section, under the subsection ‘Phase 2: Interviews – Cognitive Testing’ of the manuscript.
