# Supplementary material for: Coproducing data-driven organizational safety with patients: development and cognitive testing of a multisetting patient-reported safety concern tool
Source: Int J Qual Health Care. 2025 Jun 24;37(3):mzaf056. doi: 10.1093/intqhc/mzaf056 (PMC12246782; doi:10.1093/intqhc/mzaf056)
Supplement: mzaf056_Supplementary_Data [file mzaf056_supplementary_data.zip › Safety Tool COREQ-32checklist.docx]

**Table 3‑1 COREQ-32 checklist**

| Domain 1: Research team and reflexivity | |  |
| --- | --- | --- |
| *Personal Characteristics* | |  |
| Focus group facilitators  Interviewers | AS and NJW  AS and ATB | |
| Credentials | PhD | |
| Occupation | Research Associates (AS & ATB)  Reader in Improving Patient Care (NJW) | |
| Gender | AS (Male), NJW (Female) and ATB (Female) | |
| Experience and training | Experienced in qualitative research | |
| *Relationship with Participants* | None | |
| Relationship established | 5-10 minutes informed consent video discussion prior to the cognitive interview or focus group | |
| Participant knowledge of the interviewers/facilitators | Participants did not know the interviewers or facilitators prior to the interviews or focus groups | |
| Interviewer/facilitator characteristics | AS and ATB are trained researchers who conduct qualitative or quantitative research in areas where a need has been evidenced by their stakeholders. NJW is a Reader in Improving Patient Care with several years of experience in qualitative research. | |
| Domain 2: Study design |  | |
| *Theoretical Framework* |  | |
| Methodological orientation and theory | Content analysis. Findings, such as themes, were drawn from data collected during interviews | |
| *Participant Selection* |  | |
| Sampling | Purposive sampling of participants | |
| Method of approach | Social media platforms, newsletter of relevant charities and email | |
| Sample size | 36 Participants (phase 1=26, phase 2=10) | |
| Non-participation | None. All participants voluntarily declared interest via the eligibility screening survey. | |
| *Setting* |  | |
| Setting of data collection | Home or workplace (Online via Microsoft Teams or Zoom) | |
| Presence of non-participants | No | |
| Description of sample | Phase One (focus group)  26 participants; Ages ranged from 25–54 years (mean=32) and 23.1% were female.  Phase Two (Cognitive testing)  10 participants; Ages ranged from 25-84 years (mean=46.5) and 70% were female | |
| *Data Collection* |  | |
| Interview/ focus group guide | The questions were co-developed by the authors and a public partner. The focus group and interview guides were individually pilot tested on two volunteers. | |
| Repeat interviews | No | |
| Audio/visual recording | Audio/visual recording using MS Teams/Zoom | |
| Field notes | Notes of Peculiar situations were made after each interview or focus group | |
| Duration | focus group=60-90 minutes  cognitive testing=30-60 minutes | |
| Data saturation | Data saturation was reached when the major and minor themes were repeated in focus groups. | |
| Transcripts returned | No | |
| Domain 3: Analysis and findings |  | |
| Data analysis |  | |
| Number of data coders | One (AS) | |
| Description of the coding tree | NA | |
| Derivation of themes | Themes were derived from data collected | |
| Software | NVivo 12 | |
| Participant checking | No | |
| *Reporting* |  | |
| Quotations presented | Yes | |
| Data and findings consistent | Yes | |
| Clarity of major themes | Yes | |
| Clarity of minor themes | No minor themes identified in this study. | |
